# Early Kidney Damage Markers after Deferasirox Treatment in Patients with Thalassemia Major: A Case-Control Study

**DOI:** 10.1155/2019/5461617

**Published:** 2019-04-21

**Authors:** Hamidreza Badeli, Adel Baghersalimi, Sajjad Eslami, Farshid Saadat, Afagh Hassanzadeh Rad, Rokhsar Basavand, Soghra Rafiei Papkiadeh, Bahram Darbandi, Wesam Kooti, Ilaria Peluso

**Affiliations:** ^1^Pediatric Diseases Research Center, Guilan University of Medical Sciences, Rasht, Iran; ^2^Department of Immunology and Microbiology, Guilan University of Medical Sciences, Rasht, Iran; ^3^Cellular and Molecular Research Center, Sabzevar University of Medical Sciences, Sabzevar, Iran; ^4^Council for Agricultural Research and Economics, Research Centre for Food and Nutrition (CREA-AN), Via Ardeatina 546, 00178 Rome, Italy

## Abstract

**Background:**

The life of patients with *β*-thalassemia major depends on blood transfusion. Regular blood transfusion leads to hemosiderosis in their main organs. The aim of this study was to compare the effects of deferasirox and deferoxamine on renal damage in patients with *β*-thalassemia major.

**Method:**

The present case-control study was conducted on 60 individuals who were referred to the 17th Shahrivar Tertiary Referral Hospital in Guilan province, Iran. In this study, patients with *β*-thalassemia major who used deferasirox (*n* = 21) and patients who used deferoxamine (*n* = 19) were evaluated. The control group (*n* = 20) was selected from healthy individuals. Serum creatinine (CREA), blood urea nitrogen (BUN), and Cystatin C were measured from blood samples. Furthermore, urinary (U.) neutrophil gelatinase-associated lipocalin (NGAL), albumin (Alb), interleukin- (IL-) 18, and Kidney Injury Molecule-1 (KIM*-*1) were measured by the ELISA method and normalized for U. creatinine (CREA).

**Results:**

U. NGAL, U. IL-18, and BUN biomarkers in the deferasirox group were significantly higher than those in the control group (*p* < 0.001). U. NGAL/CREA and U. KIM-1/CREA ratios increased in both the deferoxamine and deferasirox groups compared to the control group (*p* < 0.05). U. Alb was significantly higher in patients treated with deferoxamine than in healthy participants (*p* < 0.05).

**Conclusion:**

The findings of this study indicate that after taking deferasirox, there was renal damage and an increase in inflammatory factors. Also, minor renal impairment was observed after deferoxamine administration, but it was not confirmed at the molecular level (U. NGAL and KIM-1). Therefore, it seems that patients who are taking these two drugs should be monitored carefully.

## 1. Introduction

Thalassemia is an autosomal recessive inherited disorder of globin chain production which is seen in almost all races [1]. Based on four globin chains of hemoglobin, thalassemia was categorized to the two main groups which are called alpha and beta. This abnormality is mostly observed from Mediterranean to Southeast Asian countries. Thalassemia is widespread in Iran as a part of this belt from the coastal areas of the Caspian Sea to the Persian Gulf. The prevalence of the *β*-thalassemia gene in the southern margin of the Caspian Sea is estimated at 10% [2].

The life of patients with *β*-thalassemia major depends on blood transfusion. Regular blood transfusion improves their general physical condition, and it prevents ineffective hematopoiesis complications. Repeated transfusions in these patients lead to hemosiderosis in the heart, liver, and endocrine glands. In addition to the iron deposits in various body organs, it can cause the premature death of these patients. For this reason, these patients need to use an iron chelator to remove excess iron from the body [3, 4].

In spite of a significant increase in the life expectancy of patients with transfusion-dependent anemia following the use of deferoxamine, the main cause of death in thalassemia major patients is still heart disease due to iron deposition. Studies showed that subcutaneous deferoxamine in two-thirds of the patients with thalassemia major did not prevent excess iron depletion in the heart [5]. Nevertheless, this type of administration causes local complications, which is why patients refrain from it. As a result, the need for an orally administered iron chelator drug has been felt for a long time [3, 4].

In 2005, the Food and Drug Administration (FDA) approved the usage of deferasirox to remove excess iron from blood transfusions in children over two years old [6]. Its half-life is about 12 to 16 hours, and it is mainly metabolized in the liver. Currently, it has been approved as the first oral iron chelator in more than 100 countries [7]. Digestive problems are one of the side effects of deferasirox, and symptoms include abdominal pain, nausea, vomiting, diarrhea, skin rashes, and increased liver enzymes. Moreover, kidney disease with a reduction in kidney function tends to have a severe expression [7–9].

To control renal function in deferasirox users, the levels of serum urea, creatinine, and urinary protein excretion are measured. However, recent studies have shown that serum creatinine and proteinuria levels do not accurately determine the primary renal injury stages [10]. Luo et al. [11] identified neutrophil gelatinase-associated lipocalin (NGAL) and Kidney Injury Molecule-1 (KIM*-*1) as important diagnostic markers in the early phase of acute kidney injury (AKI). In a meta-analysis, urinary interleukin (IL)-18 was also proposed as a prognostic marker for AKI [12].

As *β*-thalassemia is one of the most common diseases in Guilan province and this region, many patients with *β*-thalassemia use deferasirox for treatment. However, renal complications from deferasirox and deferoxamine, especially in the early stages, have not yet been studied in patients from this region. Therefore, the aim of this study was to compare the effects of deferasirox and deferoxamine on renal function in patients with *β*-thalassemia major by measuring early kidney damage biomarkers.

## 2. Method

### 2.1. Patients

The present case-control study was conducted on 60 patients who were referred to the 17th Shahrivar Tertiary Referral Hospital in Guilan province, Iran. In this study, patients with *β*-thalassemia major were divided to the deferasirox group (*n* = 21) and the deferoxamine group (*n* = 19) for evaluation. In addition, a control group (*n* = 20) was selected from healthy (sex and age matched) individuals. Sample size was chosen according to previous studies described in a meta-analysis [13].

### 2.2. Study Protocol

Patients aged <35 years with thalassemia major who received chelating agents (deferasirox or deferoxamine) were included in the intervention groups. Patients with major thalassemia were excluded if they used other iron chelators instead of deferasirox and deferoxamine, had multiple damaged organs due to thalassemia or other diseases such as diabetes mellitus, used drugs including corticosteroids, trimethoprim, cephalosporins, and macrolides in the last 2 weeks, and had renal or urinary tract pathology. The healthy control group was matched for age and sex. They had no infectious, autoimmune, pneumonic, nervous, and other diseases.

All patients were screened for 12 months. Patients in the deferoxamine group received this drug 5 to 7 days a week with a dose of 20-50 mg/kg per day for 12 months. Patients in the deferasirox group received this drug with a dose of 20-40 mg/kg daily for 12 months. The main doses were prescribed according to the ferritin levels in patients. All prescribed doses were in accordance with the deferoxamine and deferasirox prescribing guideline [7]. The protocol for administering deferasirox was as follows: liver enzymes were controlled monthly. If the liver enzymes were 5 times more than normal, the drug was discontinued and a weekly evaluation was performed. If the enzyme did not decrease after one month or if enzymes were reenriched after the treatment was restarted, the drug was discontinued. The levels of urea and creatinine were also evaluated monthly. If the creatinine level was greater than 33% of the creatinine level before treatment, or in two occasions reached more than the maximum range for the patient's age, the dose was halved and was controlled weekly. If the creatinine level increased or did not decrease by decreasing the dose within 4 weeks, the drug was completely discontinued. Urinalysis was done monthly. If the random protein/creatinine ratio of the urine was greater than 0.6, deferasirox was interrupted temporarily. If the proteinuria did not resolve after a month, the drug would be stopped forever.

### 2.3. Ethical Considerations

This study was performed based on to the World Medical Association Declaration of Helsinki: ethical principles for medical research involving human subjects. This study was approved by the Ethical Committee of the Vice Chancellor of Research of Guilan University of Medical Sciences (number: IR.GUMS.REC.1394.349; date: 2015-11-23). It was approved by the Iranian Registry of Clinical Trials (code: IRCT20090111001545N4). Before enrollment, the participants and their guardians were fully informed of the research purpose and written consent was obtained. They were assured that their personal information would be confidential.

### 2.4. Data Collection

Data were collected by using a two-part checklist. In the first part, demographic characteristics including the age, sex, height, and weight of patients were recorded. In the patient's history section, information on the duration of taking deferasirox was included.

### 2.5. Biochemical Assessments

For measuring serum creatinine, ferritin, blood urea nitrogen (BUN), and Cystatin C, 5 cc blood samples were collected and then serum was isolated.

Urine samples were collected in sterile polypropylene containers, centrifuged at 3000 rpm for 10 min, and stored at −80°C. Cystatin C was evaluated using the ELISA method (Bioassay Technology Laboratory, China). Creatinine and albumin (Alb) were measured using a kit (Pars Test Co., Tehran, Iran) along with an autoanalyzer (BT 2000, Italy).

Human NGAL (BioVendor Co., Czech Republic), KIM-1 (Cusabio Biotech Co. Ltd. China), and IL-18 (Bender MedSystems GmbH, Vienna, Austria) levels were measured by an enzyme-linked immunosorbent assay (ELISA), according to the manufacturer's instructions. Briefly, the cytokine in the samples is bound by the immobilized antibody in each well. An enzyme-linked monoclonal antibody specific for a given cytokine is added to the wells. Following washing, a substrate is added and the color changes in proportion to the amount of cytokine. The reaction was stopped, and the absorbance was measured based on the manufacturer's recommendation with the ELx800 brand ELISA device (BioTek Instruments Inc., Winooski, VT). The detection range for urinary NGAL, KIM-1, and IL-18 were 0.3-10 ng/mL, 0.312-20 ng/mL, and 78-5000 pg/mL, respectively. ELISA kit sensitivity for U. NGAL, KIM-1, and IL-18 were 0.02 ng/mL, 0.043 ng/mL, and 9.2 pg/mL. Furthermore, the glomerular filtration rate (GFR) was obtained using an MDRD calculator.

### 2.6. Statistical Analysis

Collected data were entered into SPSS version 16 software and descriptive data were reported using descriptive statistics (number, percent, mean, and standard deviation). The normality of quantitative data was assessed by the Kolmogrov-Smirnov test. In order to compare three groups in normal and nonnormal distributed data, ANOVA (followed by the Tukey post hoc analysis) and Kruskal-Wallis tests (followed by Dunn's post hoc analysis) were used, respectively. The chi-square test was used to examine and compare the qualitative variables.

## 3. Results

In this study, 60 individuals in 3 groups participated. They were divided into the deferasirox (21 patients, 47.6% male, age 19.48 ± 6.58 years), deferoxamine (19 patients, 52.6%, 21.26 ± 5.63), and control (20 participants, 50% male, 21.15 ± 7.27) groups, which did not differ significantly for gender distribution (*p* = 0.951) and age (*p* = 0.723). In general, 50% of the samples (30 individuals) were male.

The blood and urinary renal parameters are described in Tables 1 and 2, respectively. A comparison of the 3 groups using the ANOVA test showed higher levels of BUN (Table 1), and a comparison using the Kruskal-Wallis test showed that levels of U. NGAL and U. IL-18 (Table 2) were higher in the deferasirox group compared to the other groups (*p* < 0.001). Also, the higher levels of U. NGAL/CREA and U. KIM-1/CREA ratios in both deferoxamine and deferasirox groups compared to those of the control group were noted by the Kruskal-Wallis test (*p* < 0.05) (Table 2). A comparison between the 2 case groups showed that the ferritin level was higher in the deferoxamine group (*p* < 0.05). Urinary Alb (U. Alb) in patients who used deferoxamine was significantly higher than that in the control group (*p* < 0.05). However, the level of U. Alb in the deferasirox group was not significantly different from that in the control group (*p* > 0.05).

The abnormal percentage values of U. Alb/CREA (more than 30) in the three studied groups are compared in Table 3. These differences were not statistically significant (*p* > 0.05).

The blood and urinary renal parameters in 3 age groups are described in Tables 4 and 5, respectively. In the 25-35-year-old group, GFR (Table 4, *p* = 0.038) had a significant difference. In other age range groups, there was no significant difference (*p* > 0.05) in this parameter, whereas CREA and BUN were different in the under-18-year-old group and the 18-25-year-old group, respectively. Concerning urinary parameters, a near-significant difference of U. NGAL/CREA has been observed only in the 25-35-year-old group (Table 5, *p* = 0.05).

## 4. Discussion

Subcutaneous infusion of deferoxamine for patients with thalassemia is very painful and time consuming. Currently, using deferasirox as an oral iron chelator has been increasingly substituted because of its convenience and ease of consumption. Although there are late renal damage markers such as urine protein and albumin levels, assessing early molecular biomarkers such as NGAL, KIM-1, and cytokine IL-18 could be helpful.

The findings of this study showed that U. IL-18, U. NGAL, U. NGAL/CREA, U. KIM-1/CREA, and BUN were significantly higher in the deferasirox group than in the control group. Consistent with this study, Vichinsky et al. in the United States demonstrated that the blood creatinine level in patients who were receiving deferasirox was higher than that in healthy subjects [14]. In another study on rats, Sánchez-González et al. showed that deferasirox administration caused partial necrosis in renal tubules and increased urinary NGAL, Cystatin C, KIM-1, protein, and glucose secretion [15]. In other clinical trials, Papadopoulos et al. and Even-Or et al. also reported such complications after taking deferasirox [16, 17]. IL-18 and NGAL are secreted directly from the renal tubular cells [18, 19], and damage to the renal tubules results in the rapid secretion of these two markers. IL-18 is initially secreted from macrophages, but this cytokine is also observable in a variety of cells, including bone marrow, liver, and tubular epithelial cells, podocytes, and mesangial cells. The renal epithelia secrets NGAL in response to nephron damage, and it helps to diagnose renal damage at various clinical levels [20, 21]. Several studies have reported that an increasing level of IL-18 is an inflammatory factor in renal injury. Increases in IL-18 and NGAL levels occurred before diagnosis, and this determined the elevated creatinine or reduced eGFR levels. Buelow et al. indicated that the increase of these two markers to their peak volume was achieved 6 hours after cardiopulmonary bypass, whereas changes in creatinine or the eGFR reduction was observed at least 24 hours later [22].

In the present study, renal damage was observed due to deferasirox. In 2008, the first reported complications from this drug were observed in a 62-year-old patient with a myelodysplastic syndrome who experienced renal function reduction [23]. In 2010, Grangé et al. reported a Fanconi syndrome associated with acute renal failure [24]. The Fanconi syndrome has been reported in patients 1 to 36 months after taking this drug, and patient recovery was achieved 3-42 days after discontinuation [25]. In a study by Martin-Sanchez et al., it was found that deferasirox directly had a toxicity effect on tubular cells and it induced mitochondrial dysfunction. In this study, results showed that cell death occurred due to apoptosis as well as necrosis. It was also found that the drug toxicity depends on its action mechanism, because it depends on the iron depletion-induced downregulation of BclxL [26]. Deferasirox caused the loss of mitochondrial membrane potential, and cytochrome *c* was released from mitochondria in a time-dependent manner [26]. However, antioxidants did not inhibit the effects of deferasirox [26]. Díaz-García et al. presented a number of hypotheses in a review to explain the mechanisms of deferasirox-induced nephrotoxicity [25]. The first hypothesis was the possible transfusion of deferasirox and its intracellular accumulation in the proximal tubular cell; however, this drug has high penetration and no specific transporter has been identified. Another hypothesis suggested that deferasirox could aggregate in a tubular cell through circulation in the bloodstream, and these cells make contact with the drug by their own lumen or through the blood. Another strong hypothesis was iron deficiency. The tubular cell contains many mitochondria, which are the main iron regulators. It seems that deferasirox causes a disorder in mitochondria and toxicity actions via iron removal [25]. Sánchez-González et al. expressed its possible mechanism because of the increase in caspase 3 and DNA fragmentation [15]. However, deferasirox-induced cell death was not caspase-dependent in the study of Martin-Sanchez et al. [26]. On the other hand, iron repletion prevented BclxL downregulation, indicating that iron depletion drives deferasirox-induced BclxL downregulation and cell death [26]. Moreover, DNA fragmentation and the inhibition of cell proliferation in preclinical studies were also mediated by iron depletion and an antiproliferative effect could be inhibited in the presence of exogenous iron [27].

Due to the deferasirox complications, the European Medicines Agency (EMA) supervised this drug from 2015 [28].

In the Yew et al. and Brosnahan et al. studies, acute interstitial nephritis was reported in 2 subjects aged 62 and 70 after taking deferasirox [23, 29]. Yusuf et al. reported severe hypocalcaemia in a 43-year-old woman. The problem was eliminated after the woman stopped taking it [30]. There is no scientific evidence for this finding. However, as the age increases, the blood circulatory system and immune system become weak. Blood circulation reduction is expected to exacerbate the effect of deferasirox, and it leads to severe iron deficiency.

Another finding from the present study was the increase in ferritin and urine albumin in patients taking deferoxamine. Although this finding alone is not indicative of renal damage due to deferoxamine, the increase in U. NGAL/CREA and U. Kim-1/CREA ratios is correlated with it and it is suggestive of a complication due to deferoxamine. Failure to increase the U. IL-18, NGAL, and KIM-1 molecular factors makes it difficult to accept or reject this hypothesis. On the other hand, the comparison of the abnormal percentage of UAlb/Crea (more than 30) between the 3 groups did not show any significant difference. Although its level was higher in the deferasirox group, the deferoxamine group showed a normal amount. The deferoxamine complication hypothesis was not confirmed in this study, but a clinical study with 27 patients showed that 80% of the samples showed a reduction in GFR after deferoxamine administration [31]. In another study, Cianciulli et al. reported an impaired tubular function and an increase in urinary beta 2-microglobulin hormone levels [32]. Also, Clajus et al. reported a case of severe damage in renal tubules after taking deferoxamine in a renal transplant recipient [33]. A study by Al-Kuraishy and Al-Gareeb showed that serum ferritin levels, serum iron levels, and total iron-binding capacity in deferoxamine-treated patients were higher than those in deferasirox-treated patients; this was similar to our result. It seems that a high period of deferoxamine consumption can affect iron status in a patient during 12 months of treatment [34]. However, data from a meta-analysis suggest that a similar efficacy can be achieved depending on the ratio of doses of deferoxamine and deferasirox being compared [13]. Moreover, a major limitation of this work, according to previous suggestions, is that changes in serum ferritin may not reflect trends in iron balance equally in all patients or for all chelation regimens. From this, further studies on iron trends in tissues such as the heart are needed.

## 5. Conclusion

The findings of this study indicate that after taking deferasirox, there was renal damage and an increase in inflammatory factors. Also, minor renal impairment was observed after deferoxamine administration, but it was not confirmed at the molecular level. Therefore, it seems that patients who are taking these two drugs should be monitored carefully.

## Figures and Tables

**Table 1:** Comparison of blood renal parameters in case and control groups.

Variables	Study groups			
	Deferoxamine	Deferasirox	Control	
	Mean ± SD	Mean ± SD	Mean ± SD	*p* value^∗^
GFR	136.31 ± 27.45	123.35 ± 27.9	120.94 ± 13.32	>0.05
BUN	11.47 ± 3.1	14.86 ± 4.56^∗^	10.15 ± 2.92	<0.01^∗^
CREA	0.67 ± 0.12	0.73 ± 0.13	0.70 ± 0.17	>0.05
Cystatin C	52.88 ± 7.3	17.45 ± 5.25	36.76 ± 8.2	>0.05
GFR based on Cystatin	2.66 ± 1.72	3.33 ± 1.13	2.59 ± 1.61	>0.05

^∗^ANOVA test; GFR: glomerular filtration rate; BUN: blood urea nitrogen; CREA: creatinine.

**Table 2:** Comparison of urinary renal parameters in case and control groups.

Variables	Study groups			
	Deferoxamine	Deferasirox	Control	
	Mean ± SD	Mean ± SD	Mean ± SD	*p* value^∗^
U. NGAL	3.28 ± 1.80	13.60±8.74^∗∗^	4.04 ± 2.95	<0.001^∗∗^
U. KIM-1	2.99 ± 0.95	3.33 ± 1.32	2.93 ± 1.86	>0.05
U. IL-18	22.86 ± 7.11	69.39±23.65^∗∗^	19.32 ± 7.47	<0.05^∗∗^
U. CREA	93.89 ± 6.02	94.81 ± 7.24	95.2 ± 21.83	>0.05
U. Alb	2.94±0.06^∗∗^	1.67 ± 0.23	1.15 ± 0.53	<0.05^∗∗^
Urinary NGAL/CREA	0.08±0.01^∗∗^	0.14±0.02^∗∗^	0.02 ± 0.01	<0.05^∗∗^
Urinary IL-18/CREA	0.75 ± 0.01	0.7 ± 0.01	0.05 ± 0.03	>0.05
Urinary KIM-1/CREA	0.06±0.02^∗∗^	0.05±0.01^∗∗^	0.02 ± 0.01	<0.05^∗∗^
Urinary Alb/CREA	23.99 ± 5.8	16.65 ± 2.8	8.0 ± 2.3	>0.05

^∗∗^Kruskal-Wallis test; NGAL: neutrophil gelatinase-associated lipocalin; KIM*-*1: Kidney Injury Molecule-1; IL: interleukin; CREA: creatinine; Alb: albumin; U: urinary.

**Table 3:** Comparison of abnormal U. Alb/CREA (greater than 30) in case and control groups.

			U. Alb/CREA		Total	*p* value
			Less than 30	30 to 300		
Studied groups	Deferoxamine	Count	16	3	19	0.64
		Within studied group	84.2%	15.8%	100%	
	Deferasirox	Count	16	5	21	
		Within studied group	76.2%	23.8%	100%	
	Control	Count	5	0	5	
		Within studied group	100	0	100	
Total		Count	37	8	45	
		Within studied group	82.2	17.8	100	

Alb: albumin; CREA: creatinine; U: urinary.

**Table 4:** Comparison of blood renal parameters in 3 age groups.

			Study group			
			Deferoxamine	Deferasirox	Control	
			Mean ± SD	Mean ± SD	Mean ± SD	*p* value
Age	Under 18 years old (*n* = 18)	GFR	147.08 ± 28.40	125.12 ± 30.35	—	0.159
		BUN	9.33 ± 3.01	14.40 ± 6.19	11 ± 2.55	0.161
		CREA	0.57±0.05^∗∗^	0.66±0.13^∗∗^	0.68 ± 0.08	0.048^∗∗^
		Cystatin C	92.42 ± 112.98	17.89 ± 4.47	20.14 ± 11.10	0.593
		GFR based on Cystatin	2.37 ± 2.10	3.15 ± 0.77	3.91 ± 2.28	0.416
	18 to 25 years old (*n* = 27)	GFR	139.58 ± 27.04	130.67 ± 23.33	106.78 ± 15.78	0.053
		BUN	13.44±2.46^∗∗^	15.38±2.45^∗∗^	8.57 ± 2.37	0.002^∗∗^
		CREA	0.69 ± 0.06	0.76 ± 2.07	0.80 ± 0.16	0.103
		Cystatin C	31.01 ± 43.53	17.20 ± 7.06	59 ± 82.37	0.159
		GFR based on Cystatin	3.25 ± 1.63	3.64 ± 1.64	1.87 ± 1.55	0.166
	25 to 35 years old (*n* = 15)	GFR	112.77 ± 15.02^∗^	97.93 ± 23.16^∗^	92.09 ± 5.15	0.038^∗^
		BUN	10.25 ± 2.22	15 ± 3.61	11 ± 3.30	0.213
		CREA	0.78 ± 0.17	0.90 ± 0.10	0.94 ± 0.14	0.137
		Cystatin C	41.35 ± 36.72	16.67 ± 3.03	24.54 ± 12.12	0.233
		GFR based on Cystatin	1.76 ± 1.05	3.14 ± 0.39	2.39 ± 1.03	0.233

^∗∗^Kruskal-Wallis test; ^∗^ANOVA test; GFR: glomerular filtration rate; BUN: blood urea nitrogen; CREA: creatinine.

**Table 5:** Comparison of urinary renal parameters in 3 age groups.

			Study group			
			Deferoxamine	Deferasirox	Control	
			Mean ± SD	Mean ± SD	Mean ± SD	*p* value
Age	Under 18 years old (*n* = 18)	U. NGAL	11.22 ± 17.98	13.02 ± 18.42	2.38 ± 0.46	0.331
		U. KIM-1	2.70 ± 97	3.52 ± 1.30	2.54 ± 0.94	0.247
		U. IL-18	79.42 ± 125.14	31.34 ± 44.47	20.69 ± 36.19	0.692
		U. CREA	89.50 ± 59.74	77.40 ± 42.01	—	0.786
		U. Alb	8.25 ± 17.15	1.13 ± 1.40	—	0.571
		U. NGAL/CREA	0.11 ± 0.10	0.19 ± 0.26	—	0.664
		U. IL-18/CREA	2.11 ± 3.26	0.63 ± 1.24	—	0.386
		U. KIM-1/CREA	0.04 ± 0.03	0.06 ± 0.05	—	0.386
		U. Alb/CREA	60.48 ± 98.93	14.35 ± 16.35	—	0.828
	18 to 25 years old (*n* = 27)	U. NGAL	6.80 ± 12.99	12.01 ± 20.77	12.75 ± 22.23	0.120
		U. KIM-1	3.14 ± 1.12	3.06 ± 1.34	3.69 ± 2.98	0.583
		U. IL-18	21.28 ± 52.53	71.66 ± 158.13	21.44 ± 25.20	0.608
		U. CREA	102.22 ± 66.48	101 ± 58.34	172.50 ± 85.56	0.343
		U. Alb	0.51 ± 0.38	1.85 ± 2.97	1.22 ± 0.62	0.451
		U. NGAL/CREA	0.08 ± 0.11	0.11 ± 0.11	0.03 ± 0.02	0.442
		U. IL-18/CREA	0.15 ± 0.26	0.71 ± 1.333	0.05 ± 0.05	0.884
		U. KIM-1/CREA	0.09 ± 0.16	0.03 ± 0.01	0.02 ± 0.01	0.338
		U. Alb/CREA	8 ± 9.70	14.92 ± 18.31	9.04 ± 8.05	0.962
	26 to 35 years old (*n* = 15)	U. NGAL	3.30 ± 0.77	6.84 ± 7.38	6.06 ± 6.66	0.845
		U. KIM-1	2.91 ± 0.88	3.52 ± 1.23	2.53 ± 0.66	0.307
		U. IL-18	6.48 ± 12.24	245.53 ± 406.78	10.01 ± 13.06	0.125
		U. CREA	81.75 ± 62.45	136.33 ± 168.26	203.67 ± 98	0.317
		U. Alb	0.43 ± 0.39	3 ± 3.59	1.11 ± 0.60	0.101
		U. NGAL/CREA	0.05 ± 0.03	0.07 ± 0.04	0.02 ± 0.001	0.050
		U. IL-18/CREA	0.04 ± 0.07	0.88 ± 1.12	0.05 ± 0.09	0.153
		U. KIM-1/CREA	0.05 ± 0.02	0.06 ± 0.04	0.01 ± 0.001	0.150
		U. Alb/CREA	5.20 ± 1.65	28.92 ± 25.88	7.40 ± 2.50	0.095

NGAL: neutrophil gelatinase-associated lipocalin; KIM*-*1: Kidney Injury Molecule-1; IL: interleukin; CREA: creatinine; Alb: albumin; U: urinary.

## Data Availability

The clinical data used to support the findings of this study are restricted by the Ethical Committee of the Vice Chancellor of Research of Guilan University of Medical Sciences in order to protect patient privacy. Data are available from the authors for researchers who meet the criteria for access to confidential data.
